# Effect of Animal ByProducts Fertilization on Durum Wheat in Mediterranean Conditions: Preliminary Results

**DOI:** 10.3390/plants9091094

**Published:** 2020-08-25

**Authors:** Paolo Mulè, Marco Dettori, Gianluca Carboni

**Affiliations:** Agris Sardegna, Viale Trieste 111, 09123 Cagliari, Italy; pmule@agrisricerca.it (P.M.); gcarboni@agrisricerca.it (G.C.)

**Keywords:** animal byproducts, biofertilizing amendments, fertilization, durum wheat yield, durum wheat quality

## Abstract

This study aims to evaluate the effects of new-BioFertilizing Amendments (BFAs) deriving from fast organic matter decomposition of Animal ByProducts (ABPs) in comparison with ordinary soil organic amendments (compost), mineral N-fertilizers and no fertilization, on durum wheat development and production in a field trial under Mediterranean conditions. Results showed taller plants with heavier spikes and greater vigor in plots fertilized with BFAs when compared to no fertilization and N-fertilization, respectively. Likewise, BFAs fertilization resulted in higher protein content, gluten content, protein yields and higher values of yellow index with respect to no fertilization and N-fertilization. In contrast, lower values for test weight in correspondence of BFAs fertilization as well as no statistically significant differences on grain yield and gluten index were found. These preliminary results suggest that replacing N-fertilization with BFAs can be effective to ensure crop quality and yield stability in Mediterranean conditions.

## 1. Introduction

In order to feed the increasing world population, there is a need to produce about 50% more food by 2050. Since the 1960s, the availability of food per capita has increased more than 30% supported by a greater use of nitrogen (N) fertilizers (increase of about 800%) and irrigation water (increase of more than 100%) [1]. At the current levels of land exploitation this would require an increased cropland area between 6% and 21% more than the cropland area of 2010 (i.e., 90–325 million hectares vs 1567 million hectares) [2]. Nevertheless, intensive soil management practices (e.g., moldboard plough) as well as expanding world cropland area are bound to engender significant increases in greenhouse gas (GHG) emissions on one hand, and increasing land degradation and/or desertification particularly in the arid, semi-arid and dry sub-humid areas [3,4] on the other hand. As an outcome, increasing land overexploitation will exacerbate global warming and affect soil degradation, desertification and crop yields [1]. Concerning wheat, yields have declined at a global scale by 1.8% between 1981 and 2010 due to climate change [5]. In Europe, wheat-yield declines of 2.5% since 1989 have been reported, amplified by a drying trend leading to further yield declines of 5% or more in Italy [6]. Likewise, projected rising temperatures are expected to reduce global wheat yields by about 4%–6% for every degree of temperature rise [7,8]. Focusing on cereal–forage systems in the Mediterranean growing conditions of Sardinia, predicted durum wheat yield reductions between 16.2% and 19.0% for simulated increasing air temperature (from +1 °C to +6 °C) and decreasing rainfall (from −5% to −30%) scenarios have been reported [9]. Moreover, climate change is considered to exacerbate land degradation and potentially speed it up, owing to the joint effect of heat stress, drought and changes to evaporation rates [10], particularly in Mediterranean conditions which are projected to become drier, with the southern margins being progressively replaced by arid climate types [11,12].

Climate change, loss of arable land and declining yields have shifted the attention of public opinion to concerns such as: cultivation techniques, health of the operators and healthiness of the food, state of the environment, etc., directing the perception of crop “sustainability” towards the prevailing concepts of “environmental sustainability” and “social sustainability”. Therefore, the survival of farms cannot be separated from the economic sustainability of the crop systems.

In the long term, traditional management techniques based on deep tillage can significantly contribute to soil degradation, due to the marked alteration of the soil profile and its structure, especially if they are followed by an intensive seedbed preparation that further degrade it [13]. On the contrary, agronomic systems based on recommended management practices (RMPs), such as conservation tillage, are effective to tackle climate change since they can contribute to capture CO_2_ from the atmosphere to the soil. As an outcome, sequestering CO_2_ from the atmosphere to the soil could result in reducing CO_2_ emissions at rates between 100 and 1000 kg C·ha^−1^·year^−1^, depending on soil texture, structure, rainfall and temperature [14], as soil organic content (SOC) mineralization process is lower. Sustainable cropland management by applying these practices can improve resilience of food crop production systems to climate change and help to tackle desertification and land degradation [15,16].

Conservative agronomic systems (i.e., minimum tillage, no tillage, conservative management, etc.), have demonstrated to be an effective alternative for improving soil fertility, limiting the development of soil pathogens such as nematodes [17] and increasing yields and yield stability in Mediterranean-type soils and climate conditions [18]. In fact, an increase of one ton of soil carbon pool in degraded soils can increase crop yields by 20 to 40 kg ha^−1^ for wheat and 10 to 20 kg ha^−1^ for maize [14]. Despite the lack of updated statistics on cropland managed under conservation agriculture systems, recent estimates report a current area of 122–215 Mha (9%–15% of global arable land) and a future potential increasing adoption in the range of 533–1130 Mha (38%–81% of global arable land) [19]. In the last ten years, conservation agriculture practices have rapidly spread worldwide, including in the European Union and Italy, where these practices are supported by the rural development program of the European Common Agricultural Policy.

In addition to conservation agriculture, increasing or maintaining the same level of yield by increasing nutrient use efficiency through adoption of better fertilizer management practices, could contribute to both food security and climate change mitigation [20]. This goal may be achieved by improving nutrient, water and other input-use efficiency in order to close yield gaps, reduce GHG emissions and meet the need of food of an increasing world population [21,22,23]. The contribution of nitrogen fertilization to GHG emissions is well acknowledged. FAOSTAT (2018) indicated that cropland emissions from fertilizer application are in the range of 3.6 ± 1.2 GtCO_2_ eq*yr^−1^ over the period 2010−2016 with N_2_O emissions from synthetic fertilizers contributing for about 17% [24]. Thus, replacing chemical fertilizers with other fertilizers or amendments such as green manures, biosolids, digestate and compost can play a key role to maintain or improve soil fertility and contribute to climate change adaptation or mitigation [25].

Among the possible amendments that can be used in agriculture to foster sustainability, the use of animal byproducts (ABPs) is gaining an increasing interest. However, more specialized systems based on composting and/or anaerobic digestion are needed, and these are not yet widely used for several reasons (e.g., the compliance with the hygienic–sanitary requirements imposed by the EC regulation 1774/02; the elimination of odors in specialized systems and the N content [26]).

Sardinia (Italy) is an island located in the western Mediterranean basin. Here an innovative process for a fast non-fermentative organic matter transformation of animal and vegetal byproducts was developed and led to the creation of new soil organic amendments. This patented process (Italian patent No. 1425494, European patent No. WO2016/012986 A1, US patent 2017/0210676 A1 serial No. 15/328,512) based on a fast organic matter degradation technology allows a rapid cellular degradation of various byproducts matrices, both of animal and/or vegetal origin, resulting in a new bio-fertilizer that can be used in agriculture (Italian Ministry of Agricultural, Food And Forestry Policies - MIPAAF, Registration No. 0027872/19) and included in the class of soil amendments listed in the Italian Legislation (Annex 2 of the National Legislative Decree 75/2010).

This current study is a part of a project with the aim of evaluating the effects of these new amendments, in comparison with traditional compost, ordinary N-fertilizers and no fertilization on yield and quality of cereal–forage cropping systems of Sardinia, as well as their impact on the main physical, chemical and microbiological characteristics of soils, both on a plot scale and in open field conditions, over a 3-year time span.

In this project the new biofertilizing amendments (BFAs) are evaluated in order to assess whether they can supplement or replace chemical fertilizers in the ordinary agronomic management of the cereal–forage cropping systems of the Mediterranean basin, such that they can lead to a new model of economically competitive agriculture, more efficient in the use of resources. An approach that is more responsible towards the environment and society and, hence, fully oriented towards sustainable production.

While several studies on the influence of biowaste compost [27], composted sewage sludge [28] and biochar [29,30] on durum wheat have been carried out, to our knowledge no similar studies have been published about the fertilization effect of BFAs on durum wheat.

Here we report some preliminary results obtained during a field trial conducted in 2019.

## 2. Results

Table 1 lists the BFAs used in this study (CS/N, CS 2, CS 3, CS 4, CN), as well as their composition and application on the trial plots. The effects of BFAs on durum wheat growth and production have been compared with a binary N-fertilizer (CMIN, diammonium phosphate: N 18% and P_2_O_5_ 46%) and the control (0-treatment, U) (Table 1).

The distribution of some BFAs has determined effects on Soil Organic Matter (SOM) and wheat growth when compared to the control (0-treatment, U). Table 2 shows the effects of the different BFAs, N-fertilizer and control on the percentage content of SOM (soil sampling 0–5-cm depth), on some plant traits (i.e., plant height, spike weight and density) and on the normalized difference vegetation index (NDVI) [31], which is an index of vigor of plants, either at booting and flowering. Some BFAs treatments tend to increase the SOM content in comparison with the control. Indeed, higher values of SOM have been observed in plots treated with CS/N and CS 4 (4.31% and 3.29%, respectively) and are significantly different from 0-treatment (U), from the other BFAs CS 2 and CS 3 and from CN and CMIN.

Concerning bio-morphologic traits, the higher values of plant height and spike weight registered with ABP treatments confirm the positive effect of these amendments. In particular, all the plots treated with BFAs show a higher average plant height as well as a higher average spike weight in comparison with no fertilization and N-fertilization, respectively. The best results for plant height were registered for CS/N (73.4 cm), CN (71.3 cm), CS 2 and CMIN (70.9 cm and 70.3 cm, respectively). In contrast, untreated plots show the lowest average plant height (66.1 cm).

As for spike weight, BFAs exhibit a general positive effect with highest values for CS 2, CS 4 and CS 3 (3.69 *g*, 3.61 *g* and 3.59 *g*, respectively) while CMIN and CS/N (3.03 *g* and 2.87 *g*, respectively) are not statistically different from the untreated plots (2.61 *g*). No effects were observed on the spike density (Table 2).

**Figure 1:**
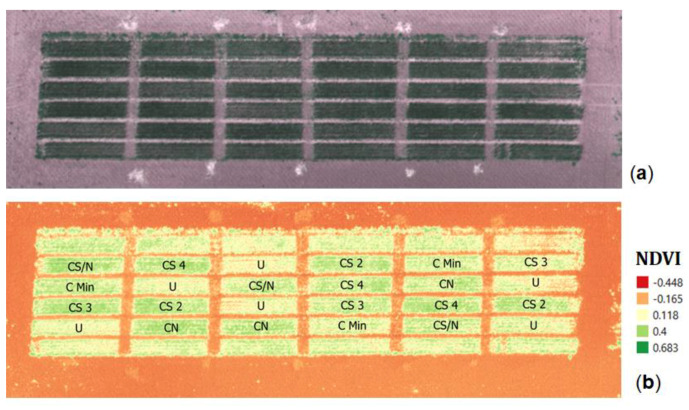
Aerial photo of the (**a**) field trial and (**b**) Normalized Difference Vegetation Index (NDVI) map at booting: higher NDVI values (dark green) are linked to a greater wheat vigor.

The effects of treatments with BFAs can also be appreciated visually in Figure 1 both from the aerial photo (a) and from the calculated NDVI map (b), where plots treated with BFAs show a darker green color corresponding to higher NDVI values. Indeed, the quantitative evaluation of the positive effect of BFAs was estimated by the normalized difference vegetation index (NDVI) either at booting or flowering.

The NDVI exhibits overall highest values in correspondence of CS 2 and CS 4 (0.412 and 0.278 for CS 2 and 0.399 and 0.277 for CS 4 at booting and flowering, respectively). These positive results are confirmed to a lesser extent for CN and CS 3 (0.384 and 0.273 for CN and 0.331 and 0.238 for CS 3 at booting and flowering, respectively). Oppositely, CMIN and U, with 0.324 and 0.217 and 0.238 and 0.121 at booting and flowering, respectively, denote the lowest NDVI values conveying less vigor compared to the other treatments.

Table 3 shows the mean comparisons of the treatments. Concerning grain yield, no statistically significant differences among treatments were found. Regarding grain quality, measured on the basis of test weight, 0-treatment (U) exhibits the best results (84.2 kg hL^−1^), followed by CMIN and CS/N (83.6 kg hL^−1^ and 83.4 kg hL^−1^, respectively). On the other hand, the lowest values were registered for CS 2 and CS 4 (82.4 kg hL^−1^ and 82.2 kg hL^−1^, respectively). On the contrary, no difference were observed for 1000 kernel weight (Table 3).

Technological quality, measured on the basis of protein content, protein yield, yellow index, gluten content and gluten index, denotes statistically significant differences among the treatments, with the important exception of gluten index. In particular, protein content and gluten content exhibit the same trend with CS 2, CS 4 and CN having the highest values (13.1% and 10.5%, 12.9% and 10.2%, 12.6% and 10.1%, respectively) while untreated plots register the lowest values (11.1% and 8.6%, respectively). Similarly, protein yield (protein content multiplied by the grain yield) shows more or less the same trend with higher values for CN (0.406 Mg ha^−1^), CS/N (0.393 Mg ha^−1^), CS 2 (0.389 Mg ha^−1^) and CS 3 (0.371 Mg ha^−1^) and the lowest one for U (0.289 Mg ha^−1^).

A positive trend among treatments for yellow index can be found in favor of BFAs: CS 3 (15.07 b*), CN (15.05 b*) and CS 4 (15.03 b*) show the highest values and CMIN and U the lowest ones (14.76 b* and 14.77 b*, respectively). In contrast, no significant trend for gluten index was found, CS/N and U showing the highest observed values (96 and 95, respectively).

## 3. Discussion

Considering the detrimental effects of soil degradation and declining yields, improved cropland management should strive to: (i) increase soil fertility by enhancing SOM; (ii) replace chemical fertilizers with green manures to restore soil fertility and preserve yield stability [6,32]. The preliminary results obtained in this study seem to confirm the effectiveness of this approach.

Concerning grain yield, data show no significant differences among treatments (Table 3). Therefore, BFAs can be a remarkable alternative to ordinary N-fertilizations. Comparing the effects of mineral fertilization with increasing doses of compost in Southern Italy, Pasqualone et al. [27] found no significant differences on durum wheat yields in correspondence of high levels of compost (12 Mg ha^−1^). Likewise, Fecondo et al. [27] found that the use of 40 Mg ha^−1^ of compost significantly increased durum wheat yields in comparison with mineral fertilization in rotation with tomato. Other studies confirm the positive effect of compost on bread wheat yields in Australia [29] and in Italy by comparing the effects of organic commercial fertilizers and compost on durum wheat yields in an organic agriculture context [30,33]. In the light of existing literature, the preliminary results presented in this study seem to open the way to the replacement of ordinary N-fertilizers with no detrimental effects on durum wheat yield, at least in the Mediterranean rainfed cereal–forage cropping systems.

Concerning the relationships between grain yield, plant height and spike weight, plots fertilized with BFAs are generally taller and exhibit heavier spikes with respect to the control and N-fertilization (Table 2). Interestingly, plants treated with BFAs show greater vigor than plants with ordinary N-fertilization as observed by NDVI measured at booting and flowering (Table 2). The positive effect on plant vigor resulting in taller plants has been described on durum wheat at increasing levels of compost application [28], although not significantly different from mineral fertilization and combined compost + mineral fertilization. Our preliminary observations highlight a general positive effect of BFAs on plant growth and development resulting in higher grain quality and protein content (Table 3). Nonetheless, this result needs being confirmed in the next cropping seasons and by further investigations due to lacking studies about this specific topic.

As for grain quality, the negative correlation between yields and test weight is well known by plant physiologists (Table 3) and is associated with the relationship between test weight and spike weight (Table 2). This result is due to the reduced competition among caryopses for the translocation of photosynthates from the stem and flag-leaf during grain-filling: fewer caryopses per spike in small spikes uptake more nutrients resulting in a larger grain size. Even though a somewhat negative effect on test weight was registered when compared with the control and N-fertilization, all plots treated with BFAs show high values of test weight, thus confirming the good to excellent grain quality (Table 3). However, a different, positive effect of compost in comparison with mineral fertilization on test weight had already been found by Fecondo et al. [27] and, to a lesser extent, by Pasqualone et al. [28].

Regarding the technological quality, protein and gluten content show significant differences among the treatments in favor of BFAs with respect to ordinary N-fertilization and no fertilization (U). This result is in agreement with Fecondo et al. [27], who found that the application of 40 Mg ha^−1^ significantly increased protein content by 26% and 14% in comparison with no fertilization and mineral fertilization, respectively. Conversely, Vaccari et al. [34] found no significant differences between the control (0-fertilization) and two biochar treatments (30 Mg ha^−1^ and 60 Mg ha^−1^, respectively) on grain N content. Noteworthy, protein yield shows more or less the same trend as protein and gluten content. Therefore, since the grain yields do not differ significantly between all treatments, the increase in protein yields is due solely to the positive effects of BFAs on the grain protein content (Table 3).

In contrast, no statistical differences among treatments for gluten index were found (Table 3), thereby confirming that technological quality does not seem to be negatively affected by replacing N-fertilization with BFAs. Interestingly, Leogrande et al. [33] found a significant difference between a commercial organic fertilizer and a compost for gluten index. However, since their study was carried out under organic conditions, it was not possible to compare the effects of biofertilizers (i.e., organic fertilizer and compost) with conventional mineral fertilization. As for yellow index of milled whole meal, a statistically significant difference among treatments was found. This result is in agreement with Pasqualone et al. [28] who found a browning effect on whole meal at increasing levels of compost application. However, the specific effect of BFAs on this quality trait must be thoroughly evaluated in the oncoming cropping seasons.

The positive correlation found both for biologic and productive data suggests that the contribution given by BFAs to the plant development can likely result in a better grain quality. Conversely, only test weight seems to be positively correlated both to N-fertilization and no fertilization with respect to BFAs. Nevertheless, given the general high values of test weight, replacing ordinary fertilization with BFAs does not seem to prevent from the large-scale use of these amendments.

## 4. Materials and Methods

This study was carried out in the AGRIS experimental farm of San Michele, southern Sardinia (Ussana, Italy) (Lat. 39°24′ N; Long. 09°05′ E; 114 m ASL) in 2019. The farm is located in the Campidano plain, a major rainfed durum wheat growing area of Sardinia.

**Figure 2:**
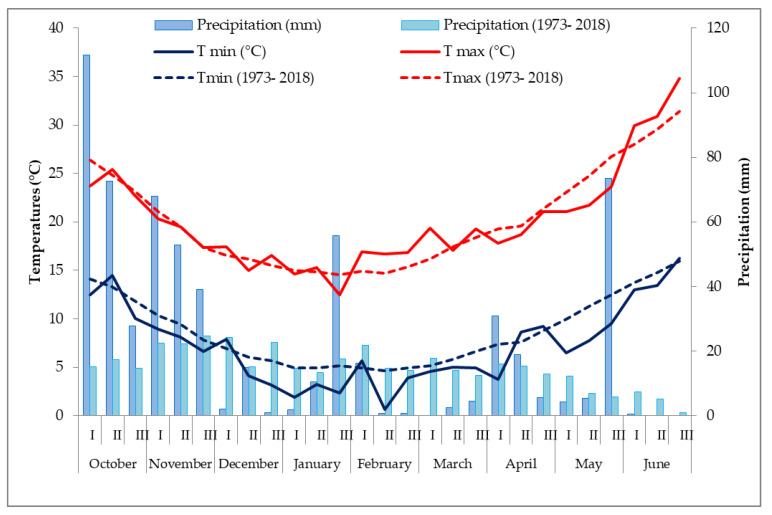
Decadal precipitations (mm), mean minimum (T min) and maximum (T max) air temperatures (°C) in the cropping season (October 2018–June 2019) and in the long-term period (1973–2018).

The climate of the study area is typically Mediterranean, with warm and dry summers and mild winters. Mean temperature values range from 4.8 °C in January to 33.0 °C in August. Total mean annual rainfall of the area is 450 mm, mostly concentrated between autumn and early spring. During the experimental period (October 2018–June 2019) we observed on average a similar trend for maximum temperatures and lower values for minimum temperatures with significant reduction of precipitation on February and March followed by heavy rain events on May when growth and development of wheat were already completed (Figure 2).

The experimental field is located in a hilly environment, typical of the low-fertility, rainfed durum wheat growing areas of Sardinia and many parts of the Mediterranean basin. The only cultivar used for this experiment is Karalis, a leading durum wheat cultivar in the last 10–15 years in Sardinia.

**Figure 3:**
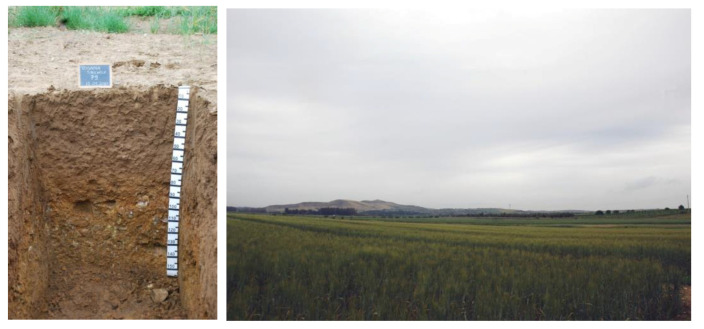
Soil profile and landscape of the area.

A pedological characterization of the site was carried out; the profile (Figure 3) was described (Table 4), samples collected and characterized [35] (Table 5).

The soil was classified as Ultic Palexeralf fine—loamy over clayey, mixed, superactive, thermic with Ap, Bw, Bt1, Bt2, Bt3 horizons [36].

The new BFAs tested in this study originate from a physical method based on the use of radio frequencies (microwaves). This non-biological process can be applied both on animal and vegetal by-products, does not provide a fermentation phase and is capable of rapidly changing the highly degradable organic biological material into a stabilized and sanitized product without risk of contamination. 

The transformation process of organic matter occurs within special machines called “fast organic matter degradation reactors” and is completed in about 1 hour, unlike the 60 − 90 days required by a natural fermentation process based on biological degradation (composting), greatly accelerating the BFA recovery and fertilizer production process. The microwave (MW) treatment acts in a dose-dependent manner and is destructive both for cells and bacterial populations, resulting in organic matter degradation (DNA denaturation) to reach a complete fast-humification. As a result, this rapid cell degradation leads to fungal reduction, bacterial, parasitological and virological inactivation (according to Annex IV, Chapter III “Standard transformation methods”, letter G, Processing method 7 of EU Regulation No. 142/2011) up to the biostabilization of organic materials.

Further, by applying such a high MW source, the electromagnetic reaction is so strong to break up in small fragments the bigger macromolecules of humic and fulvic acid chains; this modification event determines a “short chained” molecular structure phenomenon, a special structural feature that renders these molecules easier to be uptaken by the plant.

**Figure 4:**
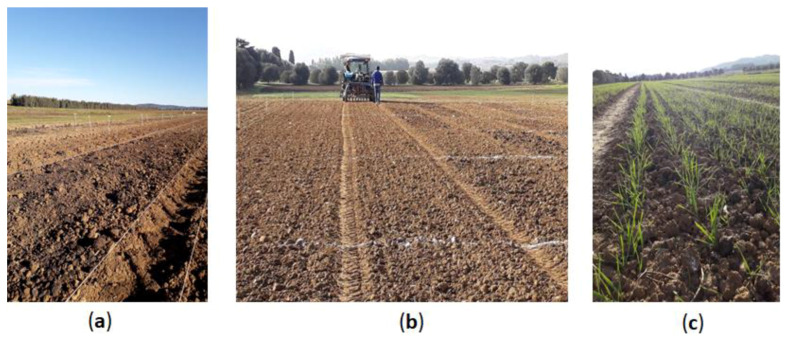
Plots during preparation. (**a**) BFAs distribution on field; (**b**) sowing; (**c**) emergence.

Treatments (see descriptions on Table 1 and chemical data on Table 6) were applied following a randomized complete block design with 3 replications. In the field trial each plot was 5.9-m-long and 1.5-m-wide with an approximate surface of 10 m^2^ and rows spaced 0.18 apart (Table 7 and Figure 4). Plant density at sowing was about 300 viable seeds m^−2^.

Before the harvest, the following biologic traits were measured to assess the effects of treatments on plant growth and development: heading date (number of days from planting), plant height (cm) and average spike weight at maturity (*g*).

Between booting and flowering, high resolution aerial imagery were collected using an Unmanned Aerial Vehicle (UAV esacopter, model HYPERION by Dronelab, Tortolì, Italy) implemented with a multispectral camera, a Parrot SEQUOIA, that can measure four bands separately: green (550 nm), red (660 nm), red edge (735 nm) and near infrared (NIR−790 nm). Aerial imagery was processed and the normalized difference vegetation index (NDVI) [31] was obtained from red and NIR bands (Figure 1) based on the following equation:

(1) $$\mathrm{DVI}=\frac{\mathrm{NIR}-\mathrm{Red}\;}{\mathrm{NIR}+\mathrm{Red}}$$

Subsequently, the NDVI map was georeferenced using an open source geographic information system [37] and sample areas, consisting of approximately 4000 pixels each, were created for each plot to evaluate the levels of wheat vigor.

Grain yield was estimated at harvest by a plot combine and, after the harvest, grain samples from each plot were analyzed to evaluate the grain quality. Protein content (%), gluten content (%), test weight (kg hL^−1^) were measured using a Foss Infratec^TM^ 1241 grain analyzer (Foss, Hilleroed, Denmark). The whole meal samples were analyzed using a color reader CR-10 Konica Minolta for color measures. Gluten index was determined following the ICC standard method No. 158 by using the Glutomatic 2200 system (Perten Instruments AB, Huddinge, Sweden). Data were statistically analyzed by ANOVA using the GenStat 19^th^ edition [38]. Multiple comparison means were made using the Fisher’s LSD method (*p* ≤ 0.05).

## 5. Conclusions

Even though based on a 1-year trial only, BFAs fertilization resulted in taller plants with greater vigor and heavier spikes as well as significant differences concerning grain quality (i.e., protein content, gluten content, protein yields and yellow index) when compared to the control (no fertilization) and N-fertilization. Furthermore, despite lower values of test weight in correspondence of BFAs fertilization were registered, all plots treated with BFAs showed high values of this trait, thereby confirming the positive effect of these amendments on grain quality. Interestingly, no statistically significant differences on grain yield were found.

These preliminary results suggest that replacing ordinary fertilization with BFAs, with or without applying conservation tillage practices, can be an effective RMP for improving soil fertility, as well as crop quality and yield stability in Mediterranean conditions.

Although these potentially positive effects, many aspects need further studies: (1) the medium and long term evolution of physical–chemical and microbiological soil fertility; (2) the medium and long term effects on the evolution of the SOM; (3) the role of different cropping systems in CO_2_ sequestration; (4) the medium and long terms effects of BFAs on durum wheat production (yield and quality) with a specific attention on technology (i.e., yellow index and protein content).

In order to give an answer to some of these open questions, BFAs will not be applied on soil in the next two cropping seasons to assess their residual effect on soil fertility as well as on durum wheat yield and quality.

## Figures and Tables

**Table 1:** Treatments applied in the field trial.

Code	Treatment	Composition	Amount (kg ha^−1^)
CS/N	Compost O.M. 1	Compost	30 × 10^3^
CS 2	Compost O.M. 2	100 kg ABP + 100 kg compost + 2 L sulfate + 50 g bio-lime + 500 mL peracid	30 × 10^3^
CS 3	Compost O.M. 3	100 kg ABP + 50 kg compost + 50 kg digestate + 2 L sulfate + 50 g bio-lime + 500 mL peracid	23 × 10^3^
CS 4	Compost O.M. 4	90 kg ABP + 45 kg compost + 45 kg potting soil + 2 L sulfate + 75 g bio-lime + 500 mL peracid	22 × 10^3^
CN	Compost nitrogen	100 kg ABP + 100 kg compost + 2 L sulfate + 50 g bio-lime + 500 mL peracid	20 × 10^3^
CMIN	N-fertilization	diammonium phosphate	200
U	Untreated		

**Table 2:** Effects of treatments on soil organic matter (SOM); plant height, spike weight, spike density and normalized difference vegetation index (NDVI) at booting and flowering.

Treatment	SOM (%)		Plant Height (cm)		Spike Weight (*g*)		Spike Density (n m^−2^)	NDVI (Booting)		NDVI (Flowering)	
**U**	2.05	**bc**	66.1	**d**	2.61	**c**	256	0.238	**d**	0.121	**c**
**CMIN**	1.63	**c**	70.3	**abc**	3.03	**bc**	278	0.324	**c**	0.217	**b**
**CN**	1.77	**c**	71.3	**ab**	3.47	**ab**	291	0.384	**abc**	0.273	**a**
**CS 2**	2.81	**bc**	70.9	**abc**	3.69	**a**	271	0.412	**a**	0.278	**a**
**CS 3**	1.99	**bc**	69.8	**abcd**	3.59	**a**	270	0.331	**bc**	0.238	**ab**
**CS 4**	3.29	**ab**	68.6	**bcd**	3.61	**a**	259	0.399	**a**	0.277	**a**
**CS/N**	4.31	**a**	73.4	**a**	2.87	**c**	288	0.391	**ab**	0.254	**ab**
*p*	0.012		0.019		<0.001		0.859	<0.001		<0.001	

Means followed by the same letter (in bold) do not differ significantly at *p* ≤ 0.05 by LSD.

**Table 3:** Grain yield and grain quality.

	Grain Yield (Mg ha^−1^)	Protein Content (%)		Gluten Content (%)		Protein Yield (Mg ha^−1^)		Test Weight (kg hL^−1^)		1000 Kernel Weight (g)	Yellow Index b*		Gluten Index (0–100)
**U**	2.61	11.1	**e**	8.6	**e**	0.289	**c**	84.2	**a**	44.8	14.77	**c**	95
**C MIN**	3.03	11.9	**cd**	9.5	**cd**	0.362	**ab**	83.6	**abc**	44.1	14.76	**c**	93
**CN**	3.22	12.6	**ab**	10.1	**ab**	0.406	**a**	82.8	**cde**	42.9	15.05	**a**	92
**CS 2**	2.98	13.1	**a**	10.5	**a**	0.389	**a**	82.4	**de**	42.0	14.96	**ab**	86
**CS 3**	3.01	12.4	**bc**	9.8	**bc**	0.371	**a**	83.3	**bcd**	43.6	15.07	**a**	94
**CS 4**	2.76	12.9	**ab**	10.2	**ab**	0.357	**a–c**	82.2	**e**	41.8	15.03	**a**	88
**CS/N**	3.34	11.8	**d**	9.5	**cd**	0.393	**a**	83.4	**abc**	43.5	14.86	**bc**	96
*p*	*0.105*	*<0.001*		*<0.001*		*0.029*		*0.004*		*0.190*	*0.004*		*0.137*

Means followed by the same letter (in bold) do not differ significantly at *p* ≤ 0.05 by LSD.

**Table 4:** Profile horizons description.

**Ap 0–40 cm**	humid; wet matrix color brown (7.5 YR 4/4); coarse / thick angular polyhedral structure, highly developed, friable when wet; few (<2%) 4 mm iron manganese nodules; 10% skeleton consisting of 10% coarse gravel (20–75 mm); small pores (0.5–2 mm) abundant (2%–5%); well drained; linear abrupt limit
**Bw 40–70 cm**	humid; matrix color from wet brown (7.5 YR 4/3); 10% of streaks of strong brown color (7.5 YR 4/6), small (<5 mm) with weak contrast; very coarse / thick sub-angle polyhedral structure, friable when wet; common (2%–20%) 1 mm iron manganese nodules; 13% of skeleton made up of coarse gravel; abundant small pores (0.5–2 mm) (0.5%–2%); well drained; linear abrupt limit
**Bt1 70–90 cm**	humid; matrix color from wet yellowish red (5 YR 5/6); 10% of streaks of yellowish red color (5 YR 5/6), medium (15 mm) with distinct contrast; multifaceted fine / thin angular structure, strongly developed, friable when wet; common (2%–20%) 4 mm manganese iron concretions and common (2%–20%) soft concentrations of 4 mm manganese iron; few films (2%–5%) of clay; 75% skeleton consisting of 50% coarse gravel and 25% pebbles (76–250 mm); abundant small pores (0.5–2 mm) (0.5%–2%); well drained; wavy abrupt limit
**Bt2 90–120 cm**	humid; matrix color from wet brown (7.5 YR 5/4); 25% of streaks of yellowish red color (5 YR 5/8), medium (15 mm) with distinct contrast and 10% of streaks of strong brown color (7.5 YR 5/6), small (<5 mm) with distinct contrast; medium-sized, highly developed polyhedral structure, highly resistant to damp; common (2%–20%) manganese iron nodules of 4 mm and common (2%–20%) soft concentrations of manganese iron of 4 mm; many clay films (15%–40%); 50% skeleton made up of pebbles (76–250 mm); small pores (0.5–2 mm) few (0.1%–0.5%); rather poorly drained; linear abrupt limit
**Bt3 120–160 cm**	humid; brown wet matrix color (10 YR 5/3); 20% of streaks of strong brown color (7.5 YR 5/6), coarse (>15 mm) with distinct massive contrast; medium-sized, highly developed polyhedral structure, highly resistant to damp; common (2%–20%) 12 mm manganese iron nodules and common (2%–20%) soft concentrations of 4 mm manganese iron; many films (15%–40%) of clay; 75% skeleton made up of pebbles (76–250 mm); very small pores (<0.5 mm) very few (<0.5%); rather poorly drained.

**Table 5:** Chemical characterization of soil samples collected from each horizon.

Horizon Code		Ap	Bw	Bt1	Bt2	Bt3
Depth	cm	0–40	40–70	70–90	90–120	120–160
Total sand	g kg^−1^	584	540	585	427	392
Total Lime	g kg^−1^	249	251	192	104	136
Clay	g kg^−1^	167	209	223	469	472
Texture class		SL	SCL	SCL	SC	C
pH H_2_O		7.4	7.9	8.2	8.1	8.3
pH KCl		5.8	6.1	6.5	6.4	6.8
Organic carbon	g kg^−1^	7.58	2.96	1.90	2.06	1.31
Organic matter	g kg^−1^	13.1	5.1	3.3	3.6	2.3
Total nitrogen	g kg^−1^	0.76	0.35	0.27	0.34	0.21
C/N		10.0	8.5	7.0	6.1	6.2
Exchangeable Ca	mg kg^−1^	1389	1563	1579	2940	2632
Exchangeable Mg	mg kg^−1^	146	178	268	628	706
Exchangeable Na	mg kg^−1^	48.6	95.6	94.6	175.2	209.6
Exchangeable K	mg kg^−1^	182	66	72	108	136
CEC	meq 100 g^−1^	15.7	16.4	19.6	24.0	33.9
Base saturation	%	56	60	54	87	60

**Table 6:** Main chemical data of treatments.

Code	Carbon %	Total Nitrogen %	C/N	Phosphatemg kg^−1^
**CS/N**	27.71	2.88	9.6	7017
**CS 2**	36.18	4.28	8.5	6927
**CS 3**	38.49	5.72	6.7	983
**CS 4**	32.42	4.46	7.3	4267
**CN**	36.18	4.28	8.5	6927
**CMIN**		18.00		46,000

**Table 7:** Main crop management information for the study period (Oct 2018–Jun 2019).

Operation	Date (mm/dd/yy)	Type
Disk harrowing (15 cm)	01/07/19	15-cm depth
Treatment fertilization	01/09/19	Table 1
Harrowing (5 cm)	01/09/19	5-cm depth
Sowing	01/17/19	Plot drill
Weed control	04/01/19	Mesosulfuron-methyl + odosulfuron-methyl-sodium + mefenpyr-diethyl + bromoxynil
Fertilization	03/13/19	Urea (60 kg ha^−1^)
Harvest	07/01/19	Plot combine
